# Age-related sarcopenia and its pathophysiological bases

**DOI:** 10.1186/s41232-016-0022-5

**Published:** 2016-09-07

**Authors:** Sumito Ogawa, Mitsutaka Yakabe, Masahiro Akishita

**Affiliations:** Department of Geriatric Medicine, Graduate School of Medicine, The University of Tokyo, Bunkyo-ku, Tokyo 113-8655 Japan

**Keywords:** Aging, Sarcopenia, Inflammation, Frailty, Hormone

## Abstract

Age-related loss of the skeletal muscle and its function is known as sarcopenia. Definition and diagnostic criteria for sarcopenia have been outlined as consensus statements from several study groups, including usual gait speed, grip strength, and skeletal muscle mass. Whereas underlying mechanisms and pathophysiology of sarcopenia remains to be clarified, recent studies have suggested that chronic inflammatory status as well as lifestyle-related factors in older individuals might contribute to the process and progress of sarcopenia.

## Background

Sarcopenia has been recently recognized as an age-related symptom which is characterized by low muscle mass, low muscle force, and low physical performance. In this review, we describe the recent progresses regarding the development of definition and diagnosis of sarcopenia, as well as its pathophysiology mainly related to age-related inflammatory processes.

## Definition and diagnosis of sarcopenia

Recent clinical and studies have suggested the presence of age-related decline in skeletal muscle mass and muscle strength from approximately the fifth decade of life, called sarcopenia [1]. This debilitating process is known to associate with frailty, disability [2], and an increased risk of fall-related fractures [3], leading to higher mortality and morbidity in the older population [4, 5]. The number of older population with sarcopenia is expected to increase all over the world, and it is becoming one of the important public concerns and interests [6].

**Fig. 1:**
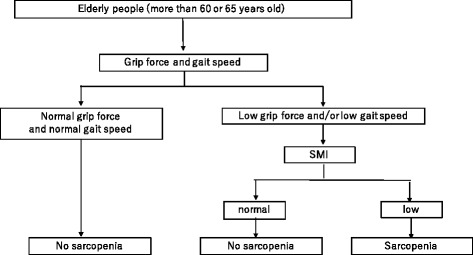
Recommended diagnostic algorithm for sarcopenia by AWGS

Sarcopenia (Greek “sarx” or flesh + “penia” or loss) was initially proposed by Rosenberg, representing age-related loss of muscle mass in its original concept [7]. Subsequently, the European Working Group on Sarcopenia in Older People (EWGSOP) defined sarcopenia in 2010 as a syndrome characterized by progressive and generalized loss of skeletal muscle mass and strength with the risk of adverse outcome such as physical disability, poor quality of life, and death [8]. The impact of sarcopenia on Asian regions including Japan is also estimated to be high, and the Asian Working Group for Sarcopenia (AWGS) agreed to describe sarcopenia as low muscle mass plus low muscle strength and/or low physical performance, further recommending its assessment in healthcare settings and in clinical practice [9] (Fig. 1). Thus, current approaches to the definition of sarcopenia are based on measurements of muscle mass, muscle strength, and functional capacity, and each indicator might be considered low when it is less than two standard deviations (2SD) away from the mean value of young male and female reference groups. The EWGSOP has developed a suggested algorithm based on gait speed measurement as the easiest and most reliable way to begin sarcopenia case finding or screening in practice.

As for the screening among community-dwelling people aged 65 years and older, the EWGSOP has developed a suggested algorithm based on (i) lower skeletal muscle mass plus (ii) lower gait speed and/or low grip strength for the diagnosis of sarcopenia [8]. AWGS also recommends using 60 or 65 years as the age for sarcopenia diagnosis according to the conditions of each country in Asia [9]. Operational sarcopenia definition by the International Working Group for Sarcopenia (IWGS) was targeted to individuals with functional decline, self-reported mobility-related difficulties, history of recurrent falls, recent unintentional body weight loss, post-hospitalization, and chronic conditions including metabolic diseases and cancer [10]. The Foundation for the National Institutes of Health (FNIH) used the data from nine sources of community-dwelling older population and proposed the cutoffs based on its analysis [11]. A comparison of definition/characteristics and cutoff values for sarcopenia in EWGSOP, AWGS, and IWGS criteria is shown in Table 1 [8–10].

**Table 1:** Comparison of definition/characteristics and cutoff values for sarcopenia by EWGSOP, AWGS, and IWGS criteria

	EWGSOP [8]	AWGS [9]	IWGS [10]
Definition/characteristics	A syndrome characterized by progressive and generalized loss of skeletal muscle mass and strength with a risk of adverse outcomes	Age-related decline of skeletal muscle plus low muscle strength and/or physical performance	Age-associated loss of skeletal muscle mass and function
SMI	7.26 kg/m^2^ for men and 5.5 kg/m^2^ for women (by DXA). 8.87 kg/m^2^ for men and 6.42 kg/m^2^ for women (by BIA)	7.0 kg/m^2^ for men and 5.4 kg/m^2^ for women (by DXA). 7.0 kg/m^2^ for men and 5.7 kg/m^2^ for women (by BIA)	7.23 kg/m^2^ for men and 5.67 kg/m^2^ for women (by DXA)
Walking speed	<0.8 m/s	<0.8 m/s	<1.0 m/s
Grip force	<30 kg for men <20 kg for women	<26 kg for men <18 kg for women	Not specified

It is proposed by the EWGSOP that sarcopenia is considered primary (or age-related) when no other cause is evident except aging itself, whereas it is considered secondary when one or more other causes are evident [8]. In practice, the etiology of sarcopenia is multi-factorial, and it might not be always possible to identify and characterize its single cause. EWGSOP also suggests a conceptual staging as severe sarcopenia, sarcopenia, and pre-sarcopenia. Severe sarcopenia is the stage when all three criteria (low muscle mass, low muscle strength, and low physical performance) are observed. The sarcopenia stage is defined as low muscle mass, accompanying either low muscle strength or low physical performance. The pre-sarcopenia stage is characterized by low muscle mass without low muscle strength or low physical performance. Evaluation of these sarcopenia stages might be helpful in light of setting appropriate recovery goals as well as selecting treatments and intervention.

In terms of epidemiology and prevalence of sarcopenia, Baumgartner et al., adopting a skeletal muscle mass index (SMI) cutoff of −2SDs below the mean of a young reference group, reported that the prevalence ranged from 13 to 24 % in persons aged 65 to 70 years old and was more than 50 % for those who were older than 80 years old [12]. Another study suggested that sarcopenia was prevalent in 10 % of men and 8 % of women older than 60 years old and that decrease in skeletal muscle was independently associated with functional impairment and disability, especially in older women [13]. The prevalence of sarcopenia in Japanese elderly men and women, based on the Asian diagnosis criteria, was 9.6 and 7.7 %, respectively [14]. The number of aged population over 60 years of age around the world was estimated to be 600 million in 2000 and is expected to rise to 2 billion by 2050. It is also estimated that sarcopenia will affect over 200 million people by the period, in contrast to the present estimation of about 50 million people [15].

## Pathophysiology of sarcopenia related to chronic inflammatory state

It is suggested that significant changes in muscle mass and its quality are observed during aging process and that there is a decrease in muscle mass at an annual rate of 1 to 2 % after about 50 years old [16]. The decline in muscle strength is supposed to be more significant, reaching to 1.5 % per year in their sixth decade and to 3 % per year afterwards [17]. In average, age-related decreases in knee extensor strength are 20–40 % compared to that of young adult mean [18], and more significant losses have been observed for those in their ninth decades [19, 20]. Recent findings suggest that multiple factors including immobility, malnutrition, low protein intake, changes in hormones and metabolism, systemic inflammation, and neuromuscular aging are supposed to influence age-related sarcopenia [21, 22].

From a histological standpoint, the skeletal muscle consists of type I and type II fibers. Type II fast fibers possess a higher glycolytic potential, lower oxidative capacity, and faster response, whereas type I slow fibers are known as fatigue-resistant due to their characteristics such as greater density and content of mitochondria, capillaries, and myoglobin. And sarcopenia is characterized by the predominant atrophy of type II fibers together with smaller and fewer mitochondria [23, 24]. Although molecular and cellular mechanisms underlying sarcopenia still remain to be clarified, age-related low-grade inflammation has been suggested to be involved as described below.

**Fig. 2:**
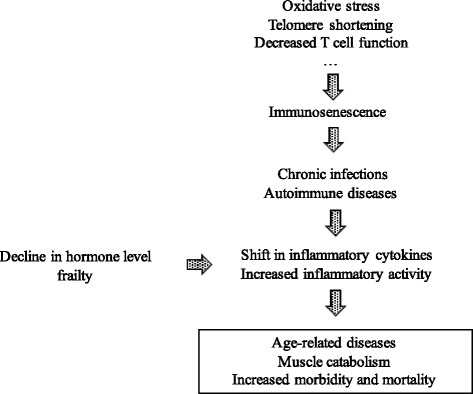
Influence of potential factors leading to age-related inflammatory processes

In general, aging is associated with a significant rise in serum levels of inflammatory markers and its related factors [25]. Franceschi et al. described the state of chronic low-grade inflammatory state as “inflammaging” based on the related concept of immunosenescence [26, 27]. Inflammation can be beneficial as an acute, transient immune response to harmful conditions including tissue injury or pathogen invasion. During aging process, these acute inflammatory responses may be impaired, leading to increased susceptibility to infection. Inflammaging is characterized as low-grade, chronic, systemic inflammation in aging in the absence of infection, which results in responses that lead to tissue degeneration. Inflammaging is also suggested to be related to various age-related diseases represented by atherosclerosis, dementia, type 2 diabetes and osteoporosis and is a highly significant risk factor for both morbidity and mortality in the elderly people [26, 28] (Fig. 2). Inflammaging is supposed to be a consequence of a reduced immune response or lifetime exposure to antigenic stimuli [29, 30], leading to the production of reactive oxygen species and tissue damage with the release of cytokines mediated by innate and acquired immune system [31]. In practice, inflammaging is accompanied by age-related decline in the number of T and B cells together with an increase of natural killer cells [32], and tumor necrosis factor-α (TNF-α), interleukin-6 (IL-6), interleukin-1 (IL-1), and C-reactive protein (CRP) are mainly involved in this process [27, 33, 34]. These cytokines are suggested to lead to a predisposition to age-related sarcopenia subsequently through the activation of the ubiquitine-protease system [35, 36]. And this altered activation of cellular signaling pathway is considered to promote the inflammatory state regardless of tissue damage or antigenic exposure, further contributing to one of the pathogenetic bases underlying sarcopenia [37–39]. It is also suggested that cytokines may antagonize the anabolic effect mediated by insulin growth factor-1 (IGF-1), involving in growth hormone resistance which limit IGF-I availability [40, 41]. Inflammaging also contributes to anabolic resistance, which is one of the main determinants of sarcopenia, implying that synthesis of skeletal muscle protein in response to physiologic stimuli is below the standard of muscle maintenance in the older population [42].

## Possible cytokines involved in age-related sarcopenia

Recent findings suggest that some inflammatory cytokines including TNF-α and IL-6 are involved in pathophysiology of age-related sarcopenia.

### TNF-α

Plasma TNF-α concentration preceded a significant decline in muscle strength at 4 years in study subjects aged 85 years [43] and in decline in muscle mass and its strength at 5 years in subjects aged 70 to 79 years at baseline [44]. Exposure of myoblasts to TNF-α causes inhibition of myogenic differentiation through increased proteolysis of MyoD by the ubiquitin-proteasome pathway in vitro [45]. TNF-α is also reported to suppress the Akt/mTOR pathway [41], promoting muscle catabolism, oxidative stress, and nitric acid production [46, 47]. Decrease of TNF-α level mediated by muscle training, for example, is suggested to cause muscle regeneration [48–50].

### IL-6

Some clinical studies including a longitudinal study in the Netherlands reported that high levels of IL-6 and CRP are associated with lower physical performance, muscle strength, and muscle mass [38, 50–58]. In a 6-year cohort of community-dwelling elderly subjects, elevated serum concentrations of IL-6 and IL-1RA have been associated with decline in physical performance [58]. Hospitalized geriatric patients with inflammation represented significantly weaker muscle function, shoulder extension strength, and a worse fatigue resistance [59]. In a cross-sectional study carried out in community-dwelling women aged more than 65 years, serum IL-6 levels were associated with higher prevalence of frailty [60]. In older women, higher serum IL-6 levels were adversely associated with recovery of lower extremity function after hip fracture [61].

In an experimental study, IL-6 transgenic mice revealed decreased skeletal muscle mass, and anti-mouse IL-6R antibody inhibited the atrophy [62]. Another study suggested that IL-6 and serum amyloid A produced in the liver synergistically increased MuRF1 and atrogin-1 expression by inducing SOCS-3 expression and impairing its downstream insulin/IGF-1 signaling in the skeletal muscle [63]. On the other hand, IL-6 is a pleiotropic cytokine, acting both as an inflammatory cytokine and as a myokine. For example, acute exercise causes skeletal muscle contraction and promotes IL-6 release into the systemic circulation, which could be beneficial for muscle growth [64]. Further studies are needed to elucidate how IL-6 are involved in the pathogenesis of age-related sarcopenia.

### Other cytokines and inflammatory substrates

A recent study suggested that IL-1 blocked differentiation of human myoblasts into myotubes by activating TGF-β-activated kinase (TAK)-1 in vitro [65] and might be involved in sarcopenia. In addition, several clinical studies imply the relationship between serum CRP concentration and sarcopenia. For example, high-sensitivity CRP levels were significantly associated with sarcopenic obesity in a Korean study [66]. Proinflammatory cytokines, such as IL-6 and TNF-α, induce the production of CRP in the liver, and it has not been clarified whether high CRP level directly affects sarcopenia.

## Conclusions

Age-related sarcopenia is a phenomenon that results in significant mortality as well as morbidity in the older population and is becoming one of the major public health problems among aging society. Emerging evidences suggest underlying mechanisms and pathophysiology of age-related sarcopenia, in which the relationship between chronic inflammatory state, muscle strength, and muscle mass seems to possess a pathogenetic basis including the control of balance between protein synthesis and its catabolism. In terms of inflammaging, age-related changes in cytokines and hormones levels are also suggested to be important risk factors for muscular impairment. A better understanding and knowledge of risk factors for sarcopenia is important to promote multidimensional approach based on its pathophysiology, defining molecular targets for intervention toward successful prevention and treatment in the near future.

## Abbreviations

CRP, C-reactive protein; IGF-1, insulin growth factor-1; IL-1, interleukin-1; IL-6, interleukin-6; SMI, skeletal muscle mass index; TNF, tumor necrosis factor
